# Assessment of probiotic and technological properties of *Bacillus* spp. isolated from Burkinabe *Soumbala*

**DOI:** 10.1186/s12866-022-02642-7

**Published:** 2022-09-29

**Authors:** Yérobessor Dabiré, Namwin Siourimè Somda, Marius K. Somda, Clarisse B. Compaoré, Iliassou Mogmenga, Lewis I. Ezeogu, Alfred S. Traoré, Jerry O. Ugwuanyi, Mamoudou H. Dicko

**Affiliations:** 1 Laboratoire de Biochimie, Biotechnologie, Technologie Alimentaire et Nutrition (LABIOTAN), Département de Biochimie Microbiologie, Ecole Doctorale Sciences et Technologies (EDST), Université Joseph KI-ZERBO, 03 P.B. 7031 Ouagadougou 03, Burkina Faso; 2 Department of Microbiology, Faculty of Biological Sciences, University of Nigeria Nsukka (UNN), Enugu state, 410001 Nigeria; 3 Département Technologie Alimentaire (DTA), Centre National de Recherche Scientifique et Technologique (CNRST) / Institut de Recherche en Sciences Appliquées et Technologies (IRSAT) / Direction Régional de L’Ouest, 03 B.P.2393 Bobo - Dioulasso 03, Burkina Faso; 4 Laboratoire de Microbiologie et de Biotechnologie Microbienne (LAMBM), Département de Biochimie-Microbiologie, Ecole Doctorale Sciences et Technologies (EDST), Université Joseph KI-ZERBO, 03 P.B. 7031 Ouagadougou 03, Burkina Faso; 5 Département Technologie Alimentaire (DTA), Centre National de Recherche Scientifique et Technologique (CNRST) / Institut de Recherche en Sciences Appliquées et Technologies (IRSAT), 03 B.P. 7047 Ouagadougou 03, Burkina Faso; 6 Laboratoire des Sciences Biologiques Appliquées, Unité de Formation et de Recherche en Sciences et Technologies (UFR-ST), Université Aube Nouvelle, 01 P.B. 234 Bobo-Dioulasso 01, Burkina Faso

**Keywords:** *Bacillus* spp., Enzymes, Probiotic, Starter, *Soumbala*, Burkina faso

## Abstract

**Background:**

*Soumbala* is a highly loved alkaline traditional fermented food condiment in Burkina Faso. It harbors various microbiota dominated by fermentative *Bacillus* spp. as functional microorganism with little confirmed health-promoting properties.

**Methods:**

The present study aimed to evaluate six *Bacillus* strains previously isolated and identified from *soumbala*. These strains were selected as presumptively safe bacteria for probiotic and technological characteristics. These strains were assessed for in vitro probiotic criteria (tolerance to acidic pH, gastric juice, 0.3% (m/v) bile salts, intestinal juice and 0.4% (w/v) phenol, cell surface hydrophobicity, auto-aggregation capacity, antimicrobial activity against foodborne pathogens, antibiotic susceptibility and biofilm production) and technological properties, including protease, amylase, lipase, and tannase activity, as well as poly-γ-glutamic acid (PGA) production and thermo-tolerance.

**Results:**

All tested *Bacillus* strains (B54, F20, F24, F21, F26 and F44) presented variable relevant probiotic properties (good tolerance to pH 2 and pH 4, gastric juice, bile salts, intestinal juice and phenol), with marked differences in hydrophobicity and auto-aggregation capacity ranging from 73.62—94.71% and 49.35—92.30%, respectively. They exhibited a broad spectrum of activity against foodborne pathogens depending on target pathogen, with the highest activity exhibited by strain F20 (29.52 mm) against *B. cereus* 39 (*p* <

0.001). They also showed good biofilm production as well as variable hydrolytic enzyme activities, including protease (43.00—60.67 mm), amylase (22.59—49.55 mm), lipase (20.02—24.57 mm), and tannase (0—10.67 mm). All tested *Bacillus* strains tolerated temperature up to 50 °C, while only strains F26 and F44 showed the best PGA production.

**Conclusion:**

Overall, the tested cultures exhibiting potential probiotic and technological characteristics; particularly *B. cereus* F20, *B. benzoevorans* F21, *B. cabrialessi* F26, and *B. tequilensis* F44 could be a source of probiotic-starters of commercial interest in the production of high-quality *soumbala*.

## Introduction

West African alkaline fermented food condiments, mainly produced by spontaneous fermentation of legumes, protein-rich seeds of cultivated and wild plant species, play major roles in the diet, socio-economic and cultural lifestyle of millions of local populations [1, 2]. Representing 80% of word food needs, fermented foods currently enjoy growing interests as functional foods [1]. Among a diversity of fermented condiments, *soumbala* is a product of the traditional alkaline fermention of *Parkia biglobosa* seeds. It is successful and widely used condiment in Burkina Faso and has various names depending on the ethnicity of local producer in other West African countries [3–5]. *Soumbala* is culturally accepted and serves as flavoring in soups, stews, spaghetti, other pastas, fried rice and chicken and constitutes a significant source of low-cost protein [3–5]. It is considered to be health-promoting, and its consumption is believed and advised as a means to prevent and/or to fight malnutrition, cardiovascular diseases among others [3, 5]. Despite rapidly changing food habits brought about by urbanization, this food seasoning has continued to enjoy sustained and growing interest, due to local preferences and increasing valorization and promotion of local foods (based on existing raw materials) as a means of fostering food security, as recommended by the Food and Agriculture Organization. Several studies have been performed in different aspects of this food seasoning, with the main prospect being the selection of technologically relevant starter cultures for the optimization of its controlled production. Results revealed that fermentation process of *soumbala* relies on indigenous microbiota predominated by *Bacillus* spp., including *B. subtilis*, *B. amyloliquefaciens*, *B. licheniformis*, *B. pumilus*, *B. megaterium*, *B. sphaericus, B. cereus, B. badius* and *B. fusiformis* [3, 6, 7]. These pre-dominant fermentative bacteria are responsible for natural bioconversion of complex food molecules, flavor (taste, texture, and aroma) development, production of antimicrobial compounds that impact shelf-life and safety, and in some instances, may confer host-beneficial health effects beyond basic nutrition. Some of these *Bacillus* strains, mainly *B. subtilis*, are recognized as technologically relevant and safe microorganisms of West African traditional alkaline fermented seed condiments [1]. Interestingly, some *B. subtilis* strains, isolated from *soumbala*, *bikalga* (fermented seeds of *H. sabdariffa*) and *maari* (fermented seeds of *A. digitata*), could be used as starter cultures for the production of high-quality *soumbala* [8]. In addition, in our previous study we highlighted that some presumptively safe *Bacillus* spp. identified by 16S RNA sequencing, could serve as potential probiotic-starter cultures [7]. However, few studies have been done on the probiotic functional properties / attributes of the relevant *Bacillus* strains from the African traditional fermented food condiments [9]. Nevertheless, some *Bacillus* strains are considered as probiotics, *i.e.*living microorganisms that, when administered in adequate amounts, confer a health benefit to the host [10]. Their potential benefits include modulation of immune system, antimicrobial activities against foodborne pathogens, reduction of cardio-vascular disease, lowering of serum cholesterol level, prevention of intestinal disorders, such as diarrhea or lactose intolerance, and of antibody-associated diarrhea [11, 12]. Probiotics have recently become available as novel foods or dietary supplements for human nutrition and as feed supplements for animal nutrition [13]. Thus, some *Bacillus* strains have been classified as Generally Recognized As Safe (GRAS) bacteria for use as foods or dietary supplements for human nutrition and as feed supplements for animal nutrition [13]. In the case of *soumbala*, to the best of our knowledge, no studies are available on functional properties of the organisms responsible for fermentation. Detailed understanding of fermentative microbiota and their unique technological and probiotic functional properties are fundamental in developing products such as *soumbala*. Therefore, this study aimed to investigate the probiotic attributes of presumptively safe *Bacillus* spp. strains isolated from *soumbala* and to advance better understanding of their role in the fermentation process for high-quality *soumbala* production.

## Materials and methods

### Microorganisms

Six *Bacillus* strains earlier isolated in our laboratory (LABIOTAN, Université Joseph KI-ZERBO, Ouagadougou, Burkina Faso) as active agents in the traditional fermentation of *soumbala* and identified through molecular biology techniques were used. These *Bacillus* strains were identified as *B. dakarensis* (B54), *B. cereus* (F20), *B. benzoevorans* (F21), *B. subtilis* (F24), *B. cabrialesii* (F26) and *B. tequilensis* (F44), with similarity / E-score of 97.51%, 100%, 97.99%, 91.58%, 100% and 97.86%, respectively in EzBioCloud^1^ [7]. All these organisms were also found to be non-hemolytic and susceptible to antibiotics currently used as medicine and then, selected as non-pathogenic strains [7]. These strains were maintained on nutrient agar slants at 4 °C prior to use. For probiotic and technology properties characterization, sterile Brain Hearth Infusion (BHI) broth was inoculated with strains and incubated at 37 °C for 24 h, referred to in this study as "24 h culture" or "overnight culture".

### Survival in different media similar to gastrointestinal tract conditions

The survival rate (SR) of the *Bacillus* strains was assessed under different conditions in media, these conditions being similar to that of the gastrointestinal tract (GIT). A 10% (v/v) aliquot of each *Bacillus* culture grown overnight in BHI broth was inoculated into 10 mL of freshly prepared BHI broth (required pH 2 or 4). A 0.1 mL of each sample was collected before (T_0_ = 0 h) and after required incubation time (T_1_ ≠ 0 h) for each test and spread onto Müller Hinton (MH) agar plates and incubated at 37 °C for 24 h.

Then, viable cells were enumerated. The relative SR of the organisms was calculated with the following formula [14]: $SR/viability=\mathrm{NT}1/NT0 x 100$, with NT_1_ = log CFU after switching to the relevant medium at t ≠ 0 h and NT_0_ = log CFU at t = 0 h.

### pH tolerance

An aliquot (1 mL) of each *Bacillus* culture grown overnight in BHI was inoculated into 10 mL nutrient broth at pH 2 and 4, respectively. A 0.1 mL of each sample was collected at T_0_ = 0 h and after incubation for 4 h (T_1_), at 37 °C. Collected samples were spread onto MH agar plates and incubated at 37 °C for 24 h [15]; then, viable cells were enumerated. The relative survival rate of the organisms was calculated using the same formula above.

### Gastric juice tolerance

An aliquot (1 mL) of each *Bacillus* culture grown overnight in BHI was inoculated into 10 mL gastric juice (2 g/L NaCl, 3.2 g/L pepsin,7 mL of 0.2 M HCl, 993 mL distilled water and pH 2) sterilized by filtration using 0.22 µm Millipore membrane filter (Easy FlowTM Filter 0.22 µm Millipore, Bedford, MA, USA). Incubation was done at 37 °C for 2 h under agitation at 150 rpm. An aliquot (0.1 mL) of each culture was collected before (T_0_ = 0 h) and after incubation (T_1_ = 2 h) and spread onto MH agar plates and incubated at 37 °C for 24 h [15]. Viable cells were enumerated; then, viability of the organisms was calculated with the same formula above.

### Bile salts tolerance

Bile salts tolerance of the *Bacillus* strains was determined using a slight modification of the method of Unban et al. [15]. An aliquot (1 mL) of each *Bacillus* culture grown overnight in BHI broth was incubated in 10 mL of freshly prepared BHI broth containing 0.3% (w/v) bile salts at 37 °C for 3 h under 150 rpm agitation. Samples were collected before (T_0_ = 0 h) and after (T_1_ = 3 h) incubation and spread onto MH agar plates using a glass rod, then, incubated at 37 °C for 24 h, after which viable cells were counted. The viability of the organisms in the BHI broth containing 0.3% bile salts was calculated using the same formula above.

### Intestinal juice tolerance

An aliquot (1 mL) of overnight culture of each *Bacillus* strain was inoculated into 10 mL intestinal juice (6.8 g / L KH_2_PO_4_ in 250 mL distilled water, 77 mL 0.2 N NaOH, 10 g pancreatin in 500 mL distilled water, make up to 1000 mL with distilled water, pH adjusted to 6.8) sterilized by filtration through a 0.22 µm Millipore membrane filter (Easy FlowTM Filter 0.22 µm Millipore, Bedford, MA, USA). The cultures were incubated under agitation at 150 rpm at 37 °C for 6 h. Then, samples were collected and spread onto MH agar plates using a glass rod, incubated at 37 °C for 24 h, after which viable cells were counted. The viability of the organisms was calculated as described above.

### Phenol resistance

The survival of the *Bacillus* strains to the action of toxic metabolites produced during digestion was determined according to the method described by Kiliç et al. [16]. Thus, 1 mL overnight culture of each *Bacillus* strain was inoculated with 10 mL sterile nutrient broth supplemented with 0.4% (w/v) phenol. Incubation was done at 37 °C for 24 h. Phenol resistance was determined according to the relationship of Burgain [14].

### Auto-aggregation

Auto-aggregation capacity of the *Bacillus* strains was determined according to a modified method of Borah et al. [17] as follows: bacterial cells were collected from 10 mL overnight culture by centrifugation at 10,000 xg for 10 min at 4 °C. The resulting cell pellets were washed twice with phosphate-buffered saline (PBS, pH 7.2) and re-suspended in 5 mL of the same buffer at 10^8^ CFU / mL. An aliquot (3 mL) of the suspension was vortexed for 10 s and incubated at 37 °C for 2 h. The absorbance of the supernatant before incubation (A_0h_) and after 2 h incubation (A_2h_) was measured at 600 nm using a UV / visible spectrophotometer. Auto-aggregation (A) was expressed using the following equation: A (%) = [(1 – (A_2h_/A_0h_)] × 100.

### Cell surface hydrophobicity

The hydrophobicity of the cell surface of the *Bacillus* strains was determined, in terms of cell's ability to adhere to hydrocarbon solvents (ACS), according to García-Hernández et al. [18]. A 24 h culture at 37 °C was centrifuged at 10,000 xg for 10 min at 4 °C. The cells were washed twice with PBS (pH 7.2), and re-suspended in 2 mL of PBS. Its absorbance was read at 600 nm and this was used as the value of A_0h_ to determine hydrophobicity in percentage. Cell suspension was mixed with equal volume of toluene, chosen as a non-polar solvent because it reflects the hydrophobicity of the cell surface [19, 20], then vortexed for 5 min. The mixture was allowed to separate into two phases at 37 °C for 1 h. The organic phase has been removed. Then, the absorbance of the aqueous phase was measured at 600 nm and used as the value of A_1h_. The hydrophobicity of the cell surface (H) or percentage of ACS was determined by the following formula: H / ACS (%) = [1—(A1/A0)] × 100. Strains with H / ACS (%) more than 50% were considered hydrophobic [15].

### Antimicrobial activity

Antimicrobial activity was evaluated against 19 pathogenic microorganisms using a cut well diffusion assay [21, 22] with some modification. These pathogens included 15 bacteria (*Bacillus subtilis* subp *subtilis* ATCC 6051, *Bacillus subtilis* subp *spizizenii*, *Bacillus cereus* 39, *Bacillus cereus* LMG 13,569, *Escherichia coli* 12, *Staphylococcus aureus* CTI, *Staphylococcus aureus* O10, *Staphylococcus aureus toxin* (A + B), *Salmonella enteritidis* P167807, *Shigella dysenteria* 370*, Pseudomonas aeruginosa* CN, *Proteus vulgaricus*, *Listeria monocytogenes* NTCT983, *Enterococcus faecalis* ATCC 19,433, and *Yersinia enterocolitica* BT3) and 4 fungal strains (*Aspergillus fumigatus, Candida albicans*, *Candida tropicalis* and *Saccharomyces cerevisiae* KVL 013) obtained from the stock culture of *Département Technologie Alimentaire* (DTA)/IRSAT. Each bacterium was grown onto BHI agar while fungal stains were grown onto potato dextrose agar (PDA). One to two colony of overnight-grown culture of each pathogen was picked up with sterile Pasteur pipette and suspended in 5 mL of physiologic saline to obtain 1.5 × 10^8^ UFC/mL (0.5 McFarland standard). An aliquot (0.1 mL) of each pathogen suspension was spread seeded onto MH agar plates (for bacteria strains) and PDA plates (for fungal strains). Wells (5 mm) were punched in the agar plates using a sterile borer. Cell free supernatant (CFS) of each *Bacillus* strain was collected from a 24 h culture by centrifugation at 12,000 xg for 15 min at 4 °C followed by filtration through a 0.22 µm Millipore membrane filter (Easy FlowTM Filter 0.22 µm Millipore, Bedford, MA, USA). Then, 50 µL aliquots of these CFS were dispensed in separate wells. The agar plates were kept at 4 °C to allow the supernatants diffuse into the agar. They were then incubated in duplicate in inverted position at 37 °C for 24–48 h (for bacteria strains) and normal position at 30 °C for 72 h (for fungal strains). The diameter of the inhibition zones around the wells was measured using a caliper.

### Biofilm production / adhesion test

The ability of *Bacillus* strains to form biofilms on food matrices or other environments was assessed as follows in two ways:

- An aliquot (10 µL) of overnight-culture of each *Bacillus* strain was inoculated into 1 mL of mannitol-casein broth (50% (w/v) each distributed in Eppendorf tubes. After incubation at 37 °C for 24 h, the bacterial inoculate were emptied and the tubes filled with a 2% (v/v) Lugol solution and kept for 30 min. Afterwards, the solution was then drained and the tubes were rinsed with tap water. The observation of a blue ring on the inner wall of the tube reflects the mediated formation of biofilms. The size and intensity of this ring was assessed on a scale [23].
- The *Bacillus* strains were grown in Luria Bertania broth (LB) (10 g/L tryptone, 5 g/L yeast extract and 5 g/L NaCl) at 37 °C for 24 h. Inocula of 2 µL and 15 µL of each strain were streaked onto LBGM agar (LB broth supplemented with 1% (v/v) glycerol; 0.1 mM MnSO_4_ and 1.5% agar) and inoculated into 15 mL of LBGM broth (LB broth supplemented with 1% (v/v) glycerol and 0.1 mM MnSO_4_) dispensed into Petri dishes, respectively. Incubation was done at 37 °C for 72 h (LBGM agar) and 24—48 h (LBGM broth). The appearance of viscous and mucoid colonies on LBGM agar or the formation of films at the LBGM broth-air interface indicate the formation of biofilms by the tested strain [24].

### Screening for technological properties

Screening of tested *Bacillus* strains for their technological properties focused on their hydrolytic enzyme activities, including protease, lipase, amylase, and tannase, and poly-γ-glutamic acid (PGA) production. The thermotolerance was also evaluated.

### Protease activity

The protease activity of *Bacillus* strains was evaluated by the spot method, using nutrient agar supplemented with 10% (v/v) skimmed milk. The appearance of transparent halos around the spots indicated proteolysis after 24 h incubation at 37 °C [25].

### Amylase activity

Amylase activity of *Bacillus* strains was evaluated by spot method, using nutrient agar supplemented with 2% (w/v) potato starch. Incubation was done at 37 °C for 24 h. Bacterial colonies grown on the agar were sprayed with Lugol solution and kept for about 15 min. The appearance of a clear halo around the colonies indicates amylase activity [26].

### Lipase activity

Lipase activity of the *Bacillus* strains was evaluated by spot method, using nutrient agar supplemented with 3% (v/v) *Cocos nucifera* oil. The appearance of clear halos around the colonies indicates lipolysis after 24 h of incubation at 37 °C [27].

### Tannase activity

Tannic acid (hydrolysable tannin) degradation was evaluated according to a modified method of Unban et al. [15]. Each strain was plated in spot on modified tannic acid agar consisting of BHI broth, 0.5% (w/v) yeast extract and 3% (w/v) agar. A 5 mL volume of filter-sterilized 2% (w/v) tannic acid was transferred to the agar surface for 15 min and the excess tannic acid solution was removed by aspiration with a sterile syringe. The opaque agar surface was washed three times with PBS solution (pH 7.2) to remove the tannic acid residue, then, an inoculum of each strain was spotted on the tannic agar surface. After incubation at 37 °C for 24 to 72 h, the appearance of a clear halo around the bacterial colony indicates the use of tannins [15, 28].

### Poly-gamma-glutamic acid (PGA) production

The detection of PGA production by *Bacillus* strains was performed on ME medium consisting of *L*-glutamic acid (20 g/L), citric acid (12 g/L), glycerol (80 g/L), NH_4_Cl (7 g/L), MgSO_4_. 7H_2_O (0.5 g/L), FeCl_3_.6H_2_O (0.04 g/L), K_2_HPO_4_ (0.5 g/L), CaCl_2_.2H_2_O (0.15 g/L), MnSO_4_.H_2_O (0.04 g/L) and agar (15 g/L) [29]. A 24 h culture of each strain in LB broth was plated on ME agar and the plates were incubated at 37 °C for 24–48 h. After incubation, the development of highly viscous and mucous colonies reflects the production of PGA by the strain [29, 30].

### Thermotolerance

A 24 h colony of each *Bacillus* strain was inoculated into 5 mL of the nutrient broth. The initial incubation was done at 45 °C for 24 h to observe growth by turbidity of the medium. After incubation, the strains that resisted the previous temperature were selected and tested at 50 °C and then at 55 °C [31]

### Statistical analyses

An analysis of variance (ANOVA) and Tukey’s mean comparison test were performed to determine significant difference (*p* < 0.05) in all activity test results using the R software version 3.6.3. The *p*-values less than 0.05 were considered to be statistically significant. Data are expressed as mean ± standard deviation of replicates.

## Results

### Survivability of Bacillus cultures in artificial media similar to gastrointestinal tract

#### Low pH tolerance

*Bacillus cereus* F20 was found to be the most acid-tolerant strain exhibiting 49.56% survivability after 3 h of incubation at pH 2, whereas *B. cabrialesii* F26 exhibited 44.40% survivability (Table 1). While at pH 4, *B. cabrialesii* F26 and *B. subtilis* F24 exhibited the highest (79.21%) and the lowest (55.90%) survival rates, respectively, after the incubation period (3 h).

**Table 1:** Relative survival rates of *Bacillus* strains in artificial media similar to GIT

**Strains**	**Survival rate (%)**					
	pH 2	pH 4	GJ	BS	IS	Phl
**F20**	49.56 ± 1.40^a^	66.11 ± 0.78^c^	44.10 ± 0.62^a^	87.91 ± 0.41^a^	70.77 ± 0.13^c^	66.96 ± 0.42^a^
**F21**	46.73 ± 0.57^bc^	66.90 ± 0.49^c^	34.55 ± 0.77^bc^	75.84 ± 0.64^d^	87.90 ± 2.55^a^	64.85 ± 0.56^a^
**F24**	44.91 ± 0.48^ cd^	55.90 ± 0.92^e^	41.27 ± 0.61^ab^	52.69 ± 0.71^f^	62.12 ± 0.77^c^	61.77 ± 0.60^abc^
**F26**	44.40 ± 1.18^ cd^	79.21 ± 0.76^a^	41.58 ± 0.80^ab^	82.03 ± 0.35^b^	88.00 ± 1.40^a^	66.98 ± 1.23^a^
**F44**	35.92 ± 0.42^ed^	69.26 ± 1.01^b^	20.55 ± 0.77^de^	60.50 ± 0.23^e^	79.62 ± 0.70^b^	61.25 ± 0.42^ab^
**B54**	48.22 ± 0.38^ab^	61.41 ± 1.15^d^	31.35 ± 1.62^ cd^	80.86 ± 0.00^c^	74.48 ± 0.57^b^	52.90 ± 0.49^bc^
***P*-value**	< 0.0001	< 0.0001	< 0.0001	< 0.0001	< 0.0001	0.02

Legend: % = percentage, *GJ*  Gastric juice, *BS*   Bile salts, *IS*   Intestinal juice, *Phl*  Phenol; means ± standard error; values with different superscript letters in the same column are significantly different (*p* < 0.05)

#### Gastric juice tolerance

The *Bacillus* cultures showed relative survival rates range of 20.55–44.10% after exposure to gastric juice for 3 h (Table 1), with *B. cereus* F20 showing the highest survival rate while *B. tequilensis* F44 showed least survival rate.

#### Bile salts tolerance test

Results showed that *B. cereus* F20 was highly bile tolerant, maintaining 87.91% viability while *B. subtilis* F24 maintained 52.69% viability after 3 h incubation in BHI containing 0.3% (w/v) bile salts (Table 1).

#### Phenol tolerance

In BHI broth containing 0.4% phenol, *B. cabrialesii* F26 displayed the highest viability / survivability (88%) while *B. dakarensis* B54 demonstrated lowest viability (52.90%) after 24 h of incubation (Table 1).

### Cell surface hydrophobicity and Auto-aggregation

**Fig. 1:**
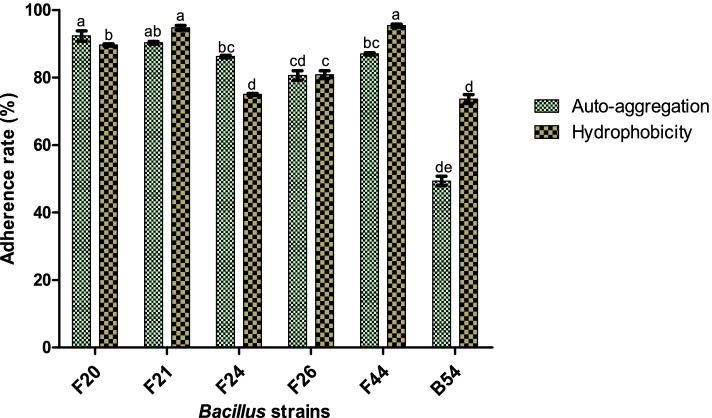
Hydrophobicity and auto-aggregation ability (%) of the studied *Bacillus* strains with toluene. Values with different letters indicate significant differences by Tukey's test (*p* < 0.05)

*Bacillus tequilensis* F44 showed the highest surface hydrophobicity (95.33%) and *B. dakarensis* B54 had the lowest value (73.62%). Regarding auto-aggregation ability, a marked difference in adhesion from 73.62 to 95.3% was observed among all *Bacillus* strains tested ( Fig. 1).

#### Antimicrobial activity

The antimicrobial activity of the neutralized cell-free crude supernatant (pH 7.0) of all *Bacillus* strains was evaluated against 19 pathogenic and potentially microorganisms. The antimicrobial spectrum obtained varied according to the test *Bacillus* strain (Table 2). Thus, *B. benzoevorans* F21 showed the broadest antimicrobial spectrum against 10 bacteria out of the 19 pathogens while *B. cereus* F20 exhibited the largest inhibition diameter (29.52 mm) against *B. cereus* 39 with a very high significant difference (*p* ≤ 0.0001). However, all fungal strains in addition to 5 bacterial strains (*E. coli 12*, *S. aureus* O10, *S. dysenteria* 370, *S. enteridis* and *Y. enterocolitica* BT3) were resistant to the inhibitory effect of the crude supernatant of all *Bacillus* strains studied.

**Table 2:** Antimicrobial activities of *Bacillus* isolates

**Indicator** **strains**	**Inhibition diameters (mm) of pathogens growth by *Bacillus* strains**						*P-* value
	F20	F21	F24	F26	F44	B54	
*B. cereus 39*	29.52 ± 1.78^a^	28.91 ± 4.82^ab^	22.23 ± 2.45^abc^	21.82 ± 0.45^abc^	18.27 ± 4.29^cb^	10.57 ± 0.81^c^	< 0.0001
*B. cereus* LMG 13,569	27.36 ± 0.19^a^	20.67 ± 0.24^b^	22.23 ± 3.01^b^	17.19 ± 0.55^c^	27.72 ± 0.31^a^	0.00 ± 0^d^	< 0.0001
*B. subtilis* subsp. *subtilis* ATCC 6051	13.61 ± 0.15^b^	12.16 ± 1.18^c^	10.06 ± 0.08^d^	11.11 ± 0.16^ cd^	15.52 ± 0.03^a^	12.00 ± 0^c^	< 0.0001
*B. subtilis* subsp. *spizizenii*	0.00 ± 0^b^	12.25 ± 0.35^a^	0.00 ± 0^b^	0.00 ± 0^b^	0.00 ± 0^b^	0.00 ± 0^b^	< 0.0001
*L. monocytogenes* NTCT983	21.57 ± 2.72^a^	23.32 ± 2.36^a^	21.65 ± 0.91^a^	25.39 ± 1.96^a^	21.64 ± 0.19^a^	11.93 ± 0.86^b^	< 0.0001
*Ent. faecalis* ATCC 19,433	23.93 ± 0.78^b^	25.53 ± 1.00^a^	17.43 ± 0.12^c^	0.00 ± 0^d^	22.49 ± 0.69^b^	0.00 ± 0^d^	< 0.0001
*S. aureus toxin* A + B	0.000 ± 0^d^	9,25 ± 0.35^c^	11.41 ± 2.00^c^	16.81 ± 0.35^a^	14.25 ± 1.06^b^	0.00 ± 0^d^	< 0.0001
*S. aureus* CTI	26.50 ± 2.12^a^	28.16 ± 2.83^a^	19.08 ± 1.38^b^	0.00 ± 0^c^	20.00 ± 2.82^b^	0.00 ± 0^c^	< 0.0001
*P. aeruginosa* CN	29.00 ± 1.41^a^	20.38 ± 0.51^c^	16.50 ± 0.70^d^	11.72 ± 0.84^e^	26.00 ± 1.41^b^	0.00 ± 0^f^	< 0.0001
*P. vulgaricus*	27.09 ± 1.28^a^	27.50 ± 0.70^a^	17.50 ± 0.70^b^	0.00 ± 0^c^	27.19 ± 1.40^a^	0.000 ± 0^c^	< 0.0001
*E. coli* 12	0.000	0.000	0.000	0.000	0.000	0..000	-
*S. aureus* O10	0.000	0.000	0.000	0.000	0.000	0.000	-
*S. dysenteria* 370	0.000	0.000	0.000	0.000	0.000	0.000	-
*S. enteridis* P167807	0.000	0.000	0.000	0.000	0.000	0.000	-
*Y. enterolitica* BT3	0.000	0.000	0.000	0.000	0.000	0.000	-
*C. albicans*	0.000	0.000	0.000	0.000	0.000	0.000	-
*C. tropicalus*	0.000	0.000	0.000	0.000	0.000	0.000	-
*S. cerevisiae KVL 013*	0.000	0.000	0.000	0.000	0.000	0.000	-
*A. fumugatus*	0.000	0.000	0.000	0.000	0.000	0.000	-

Legend: Mean ± standard deviation; values with different superscript letters in the same row are significantly different (*p* < 0.05)

**Fig. 2:**
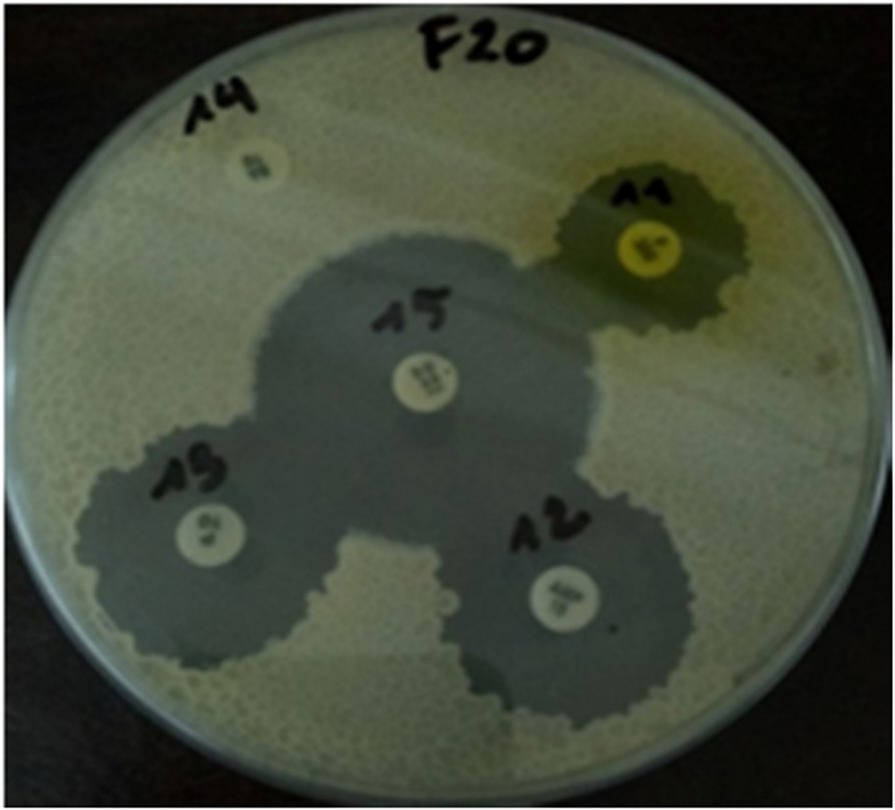
Antibiotic susceptibility of *B. cereus* F20

Regarding antibiotic susceptibility, the results of our previous study showed that all tested *Bacillus* strains were susceptible to almost all antibiotics except bacitracin for which they were all resistant. *Bacillus benzoevorans* F21 and *B. cereus* F20 (Fig. 2) were the most sensitive to imipenem (38.80 ± 1.57 mm and 38.04 ± 1.73 mm, respectively) while *B. dakarensis* B54 displayed the weakest sensitivity to bacitracin (11.00 ± 0.63 mm) [7]

#### Detection of biofilm formation

**Fig. 3:**
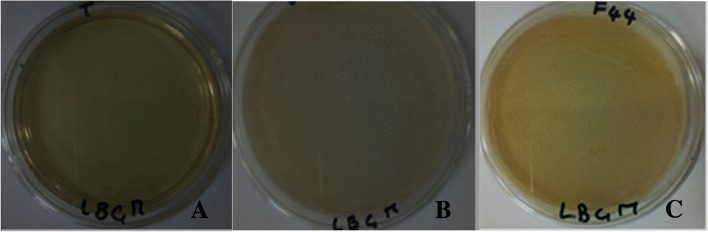
Production of biofilm as a veil on the surface of LBGM by *B. cabrialesii* F26 and *B. tequilensis* F44. Legend: **A** = Control, **B** = *B. cabrialesii* F26 and **C** = *B. tequilensis* F44

*Bacillus* species generally produce biofilms under harsh living conditions. This biofilm production depends on the microorganism and the culture conditions. All *Bacillus* strains tested in this study were able to produce different biofilm forms of varying thickness and density on the surface of the LBGM medium and on the inner wall of the Eppendorf tubes. *Bacillus* *cabrialesii* F26, and *B. tequilensis* F44 showed the best biofilm production at the LBGM broth-air interface (Fig. 3) and on the inner wall of the Eppendorf tube.

### Technological properties: Protease, amylase, lipase, tannase, and PGA production and thermotolerance

**Fig. 4:**
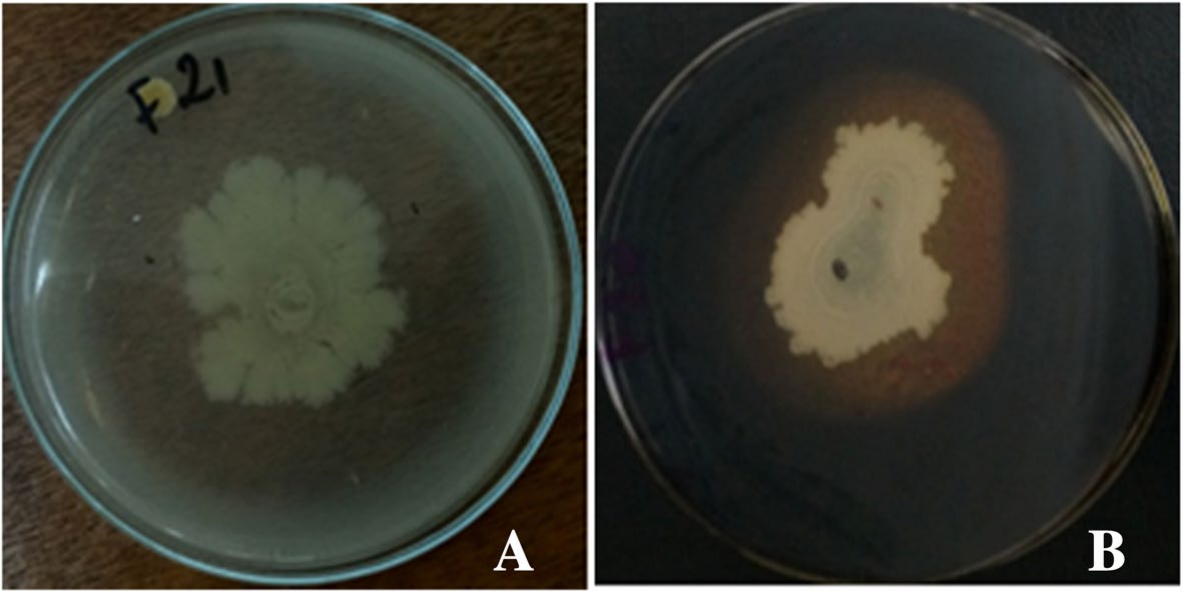
Enzymatic activity of the studied *Bacillus* strains. Legend: **A** = protease activity of *B. benzoevorans* F21, **B** = amylase activity of *B. cereus* F20

The occurrence of certain key hydrolytic enzymes activities including protease, lipase and amylase were found in all *Bacillus* strains tested. The activity diameters obtained following the expression of these enzymes varied among strains (Table 3). Thus, all *Bacillus* strains expressed various protease, amylase, lipase, and tannase activity. The highest proteolytic, amylolytic, and lipolytic activities were observed with *B. benzoevorans* F21 (60.675 mm, Fig. 4A), *B. cereus* F20 (49.55 mm, Fig. 4B), and *B. benzoevorans* F21 (24.57 mm) and *B. subtilis* F24 (24.57 mm), while the lowest activities were observed with *B. dakarensis* B54 (43.00), *B. subtilis* F24 (22.59 mm) and *B. tequilensis* F44 (20.025 mm), respectively. For tannase production the entire *Bacillus* strains where able to grow on the tannic acid medium. However, only *B. cabrialesii* F26 and *B. tequilensis* F44 showed (the lowest (09.87 mm) and highest (10.67 mm)) tannase activity diameter, respectively (Table 3).

**Table 3:** Enzymatic activity of *Bacillus* strains

***Bacillus Strains***	**Enzyme activity (diameter of clear zone in mm)**			
	**Protease**	**Amylase**	**Lipase**	**Tannase**
F20	52.60 ± 1.27^b^	49.55 ± 0.77^a^	23.25 ± 1.76^a^	0 ± 0^b^
F21	60.675 ± 0.10^a^	44.5 ± 0.70 ^b^	24.57 ± 0.67^a^	0 ± 0^b^
F24	50.50 ± 0.70^bc^	22.59 ± 0.57^d^	24.57 ± 0.67^a^	0 ± 0^b^
F26	47.00 ± 2.12^ cd^	33.85 ± 0.21^c^	22.625 ± 0.67^a^	9.87 ± 2.65^a^
F44	49.00 ± 1.41^bcd^	34.16 ± 0.25^c^	20.025 ± 0.10^b^	10.67 ± 0.95^a^
B54	43.00 ± 1.41^de^	24.725 ± 1.80^d^	24 ± 0.21^a^	0 ± 0^b^
*P*-value	< 0.0001	< 0.0001	< 0.013	< 0.0001

Legend: Mean ± standard deviation; values with different superscript letters in the same column are significantly different at < 0.05

**Fig. 5:**
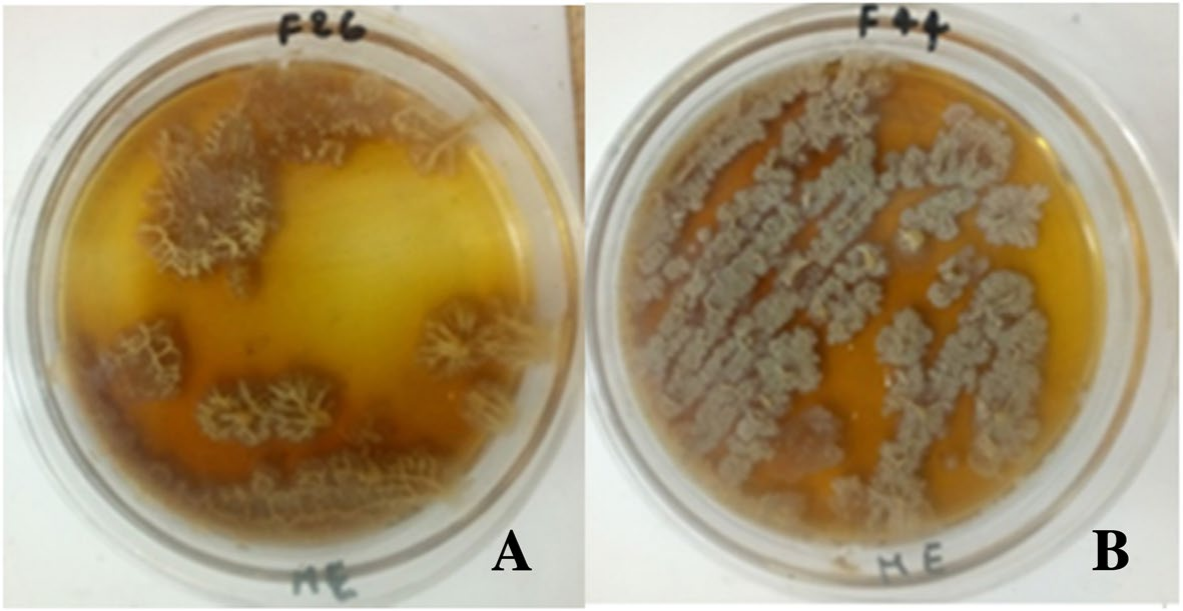
Production of PGA by *Bacillus* strains. Legend: **A** = *B. cabrialesii* F26 and **B** = *B. tequilensis* F44

Out of the 6 *Bacillus* strains tested, 3 strains, *i.e., B. subtilis* F24, *B. cabrialesii* F26 and *B. tequilensis* F44 were capable of producing PGA and this was evidenced by the shape of their highly viscous and mucous colonies on the ME (Fig. 5).

## Discussion

This study assessed for the first time the probiotic potential of *Bacillus* strains isolated from *soumbala,* a very prized food condiment. This flavoring agent production is mainly assured by *Bacillus* species responsible of macromolecules bioconversion into assimilable metabolites and production of biomolecules with a crucial role on the organoleptic (including flavor) and nutritional quality, and bioconservation of product with functional properties [3, 6, 7].

Indeed, the recognized GRAS status of several *Bacillus* strains increased their interest as probiotic-starter cultures for the development of functional foods having probiotic benefit on consumers. However, before probiotic strains are able to exert their beneficial effects in the host gut, they need to remain alive during both ingestion and in the harsh environments of the gastrointestinal tract, which include the acidic condition of the stomach. The survivability of *Bacillus* spp. in the gastric juice depends on their ability to tolerate low pH, which is an important probiotic characteristic [20, 32]. It was observed that all *Bacillus* cultures were able to withstand acidic conditions (Table 1). Similar heterogeneity in response to acidic environments was previously reported within the *Bacillus* species. Elsewhere, after exposure to low pH solution (pH 2.0, and 4.0) for 3 h, probiotic *Bacillus* strains, MKSK-E1, MKSK-J1, and MKSK-M1 showed relative survival rates of 93.1%, 91.9%, and 96.0%, respectively [33]. *Bacillus licheniformis* Me1, *B. flexus* Hk1, and *B. subtilis* Bn1 were also found to present survival rates ≥ 80% at pH 3 [32] higher than our current finding. Maintaining viability after exposure to the acidic environment for 3 h implies the strain’s ability to favorably readjust in the acid-stressed environment and resume growth. This could be by a combination of genetic and physiological mechanisms, common with acidophilic microorganisms. Acid tolerant strains are also most likely to benefit from acid protection effect of high protein and high fat diets and thus confer health benefit on consumers.

In gastric juice, all *Bacillus* strains showed survival rates ranging from 20.55—to 44.10% after exposure for 3 h (Table 1). This is less than that reported by Lee et al. [33] for probiotic *Bacillus* strains, MKSK-E1 (93.1%), MKSK-J1 (91.9%) and MKSK-M1 (96.0%) after exposure to 0.1% pepsin solution (pH 2.0) for 3 h. Acid stability is an important parameter and a basis for the selection of a probiotic strain since acid resistance is an indication of the potential of the probiotic strain to survive the gastric and duodenal juices [34].

Bacteria growth is inhibited by bile which enters through the duodenal section of the small intestine; this is possible as the bacteria cell membrane is made up of lipids and fatty acids which are sensitive to bile salts [9]. However, resistance to these substances is of great importance in survival and growth of bacteria in the intestinal tract and thus it is a pre-requisite for the selection of probiotic strains [32, 34]. Bile tolerance studies are mostly carried out using 0.3% oxgal bile solution because of its similarity to human bile juice [35] and also because 0.3% is considered to be a crucial concentration to evaluate a bile-tolerant probiotic [36]. It was observed that, *B. cereus* F20, *B. benzoevorans* F21, *B. cabrialesii* F26, *B. tequilensis* F44 and *B. dakarensis* B54 were tolerant to 0.3% oxgal bile, while *B. subtilis* F24 was weakly tolerant, suggesting that all these *Bacillus* strains are member of "tolerant" group. This result is in agreement with those observed with others *Bacillus* spp. [32, 37]. However, the survival rate obtained in the present study are lower than those reported by Kavitha et al. [20] on *Bacillus* strain FC6 which viability was 91.62%, after 3 h exposure to 1% (m/v) bile salts. The current findings are an indication that these bacteria when consumed with the fermented food have the potential to survive the acid-and bile-rich environments, a pre-requisite necessary to reach and survive in the intestinal gut in order to confer its benefits to the host [9].

Phenols are toxic metabolites which are released during digestion by bacterial deamination of some aromatic amino acids derived from dietary and endogenous proteins. These compounds are known to have bacteriostatic properties [38]. In contrast to the high phenol (0.4%) resistance, which was previously reported for *B. cereus* strains [9], the *Bacillus* spp. strains in this study were highly sensitive towards this compound, and this, despite the fact that these bacteria are physiologically closely related. Our results on the phenol resistance of *Bacillus* strains suggest that these are generally moderately tolerant. Hence, bacteria tolerant to phenols may have more chances of survival than those which are not [38], meaning that a potential probiotic strain should tolerate the limited amounts of phenols in the gastrointestinal tract.

Hydrophobicity is an important feature which aids the attachment of probiotic microorganisms to the intestinal epithelium [39]. Probiotic microorganisms, through their adhesion capability, can prevent pathogens access by steric interactions or specific blockage of cell receptors [20]. The tested *Bacillus* strains have presented variably high hydrophobicity and auto-aggregation ability (Fig. 1). This finding suggests that all *Bacillus* cultures have increased level of adhesion and colonization ability, which can prevent pathogenic access by steric interactions or specific blockage of cell receptors [40]. The difference in the level of adhesion among the tested cultures could be attributed to several factors such as the non-specific reaction by charge and hydrophobicity [32]. Moreover, hydrophobicity capacity of all *Bacillus* strains tested in toluene was remarkably higher than that of *B. cereus* strains, BC1 (52.8%), and BC2 (58.5%) from Nigerian *daddawa* [9], *Bacillus* spp. MKSK-J1, MKSK-E1, and MKSK-M1 from Korean traditional soy sauce with values less than 35% [33]. However, our results are in agreement with those observed by Talebi et al. [41] for *Bacillus* strains, 437F (57.4%), 1630F (98.0%), and 1020F (83.7%). Cell surface hydrophobicity increases the propensity of microbial cells to adhere to surfaces and this adhesion capability is the primary stage in microbial colonization, making the cell surface hydrophobicity a crucial property in cell attachment to surfaces [42]. Auto-aggregation and cell surface hydrophobicity are directly correlated, and according to Manhar et al. [43], hydrophobicity could be one of the factors that determine the ability of bacterial cultures to auto-aggregate. Regarding auto-aggregation test, *B. cereus* F20 showed the highest value (92.30%) whereas *B. dakarensis* B54 exerted the lowest value (49.35%) for 3 h incubation (Fig. 1). The auto-aggregation rates of our *Bacillus* strains were higher than that reported by Nwagu et al. [9] for probiotic *B. cereus* strains, BC1 (53.7%), and BC2 (48.69%) and by Talebi et al. [41] for *Bacillus* strains, 437F (23.2%), 1630F (20.5%), and 1020F (38.8%) for 3 h. However, auto-aggregation rate of the *Bacillus* strains tested fit in with results obtained by Manhar et al. [43] for *B. amyloliquefaciens* strains (65.5–75.5%) for 24 h, and by Lee et al. [33] for *Bacillus* strains, MKSK-E1, MKSK-M1, and MKSK-J1 with both 90% auto-aggregation rates. Auto-aggregation ability is related to the ability of the microbial cells to adhere to the gut epithelial cells [44], a key factor in microbial colonization and persistence in the host’s gastrointestinal tract [9] where they exerted antimicrobial effect against pathogens.

Previous studies reported the antimicrobial activity of numerous *Bacillus* strains isolates from various plant-based traditional fermented food condiments in Africa. A wide antimicrobial spectrum of *Bacillus* spp. isolated from *Bikalga* has been reported by Compaoré et al. [45]. The anti-fungal activity of *Bacillus* spp. isolated from *Maari* has also been reported by Kaboré et al. [46]*.* However, this was not observed in the current study. It is well documented that the antimicrobial spectrum depends on the used method, the nature and/or concentration of antimicrobial compounds or the nature of indicator pathogens [45]. Thus, the current finding could be explained by the above raisons. The ability to produce antimicrobial compounds is one of the key characteristics used to assess the probiotic potential of bacteria [47, 48]. Their secondary metabolites are involved in defense against pathogens through the cleaving of their receptor sites in intestinal epithelial cells [44]. Among various antimicrobial compounds produce by *Bacillus* species, bacteriocins such as subtilisin, subtilosin are mainly recognized to help digestion and reduce allergenicity.

For antibiogram, our previous study revealed that all *Bacillus* strains were susceptible to the majority of antibiotics except bacitracin for which they were all resistant, suggesting that these *Bacillus* strains may not carry antibiotic resistance genes that can be transferred to pathogenic microorganisms [7]. Moreover, our findings were in line with similar previous reports on the susceptibility of *Bacillus* species to several antibiotics commonly used in medicine [9, 20, 21, 49].

Regarding biofilm production by *Bacillus* strains, our results were similar to those reported by Latorre et al. [23] and Shemesh and Chaia [24]. Although biofilms are involved in the majority of chronic infections [50], conversely, they have roles in biocontrol processes [51]. Indeed, the ingestion of food containing *Bacillus* spores orally and the germination of these spores in the intestinal tract allow the proliferation of vegetative cells that adhere as a biofilm and colonize the surface of the intestinal mucosa [39]. Thus, these biofilms help *Bacillus* to attach to the epithelial cells of the intestine [52] and increase their persistence and proliferation on the intestinal mucosa where they prevent the adhesion of entero-pathogens [23] and exert the probiotic effects to host [39]. *Bacillus* are being explored for their probiotic potential in animals and humans [13]. Studies comparing *Bacillus*-containing foods to *in-vivo* standards have reported numerous health benefits of the latter [53]. These beneficial effects include modulation of the gastrointestinal tract, activation of macrophages, aggregation with pathogens, intestinal barrier function, restoration of intestinal flora, anti-inflammatory and anti-cancer activity, reduction of blood and heart disease [54, 55]. Thus, the field of investigations on *Bacillus* has recently focused on their probiotic [13, 52] and therapeutic [55, 56] applications. Indeed, several studies have shown that *Bacillus* strains can be used in the treatment of diarrhea, reduction of cholesterol levels, etc. [15, 54, 57, 58]. This has allowed some *Bacillus* strains such as *B. clausii*, *B. coagulans*, *B. licheniformis* and *B. subtilis* to be included in the Food and Drug Administration (FDA) list of so-called GRAS bacteria [13].

The current results on the evaluation of the technological properties showed that all *Bacillus* strains used in this study have very interesting enzymatic background through protease, amylase, lipase, and tannase activity (Table 3, Fig. 4A and B), PGA production (Fig. 5), and thermotolerance. For a probiotic strain to effectively function as a food fermenter, the synthesis of these hydrolytic enzymes is required to break down the complex food polymers in order to generate simpler compounds such as peptides, amino acids, reducing sugars, and oligosaccharides which will be further converted through other biological reactions to organic acids and other flavor-impacting and health benefiting compounds [59]. Hence, through these enzymatic activities, *Bacillus* degrade poorly digestible or non-digestible and toxic macromolecules and anti-nutrients into assimilable metabolites and produce biomolecules with a crucial role on the organoleptic and nutritional quality of fermented foods [60, 61]. For example, they hydrolyze casein to peptides and amino acids, polysaccharides to simple carbohydrates, lipids to fatty acids [3, 15, 62, 63] and tannins to glucose and assimilable gallic acid [15]. The hydrolytic by-products of these enzymes also engage in biological and chemical reactions to produce flavor compounds which give the fermented food its characteristic properties. The metabolism of anti-nutritional factors by fermentative micro-organisms plays a crucial role in improving the nutritional quality of fermented grain-based foods [64]. This ability to metabolize tannins depends on the type of micro-organism and the culture conditions [15]. Thus, all *Bacillus* strains tested in the current study were found to be hydrolysable tannin tolerant and thus able to grow on tannin agar medium. However, only *B. cabrialesii* F26 and *B. tequilensis* F44 were able to metabolize tannic acid. For PGA production, *B. subtilis* F24, *B. cabrialesii* F26 and *B. tequilensis* F44 were able to produce PGA as viscous and mucous colonies on ME medium. The production of PGA by *B. subtilus* strains had also been reported by previous investigators [29, 30, 65]. This polymer is used in medicine, food, etc. due to its excellent properties of biodegradability and non-toxicity to humans [66, 67]. The production of PGA by *Bacillus* strains improves the organoleptic and nutritional quality of fermented products. Through the enzymatic activities and antimicrobial metabolites production potential, *Bacillus* species participate either directly or indirectly in the development of organoleptic and nutritional quality and safety of fermented products [3, 26, 46, 68]. Todays, *Bacillus* strains (mainly *B. subtilis*) are being explored as starters to guarantee the quality of traditional African fermented foods [1, 3, 69].

Overall, the evaluation of probiotic and technological characteristics of *Bacillus* strains in the current study highlighted their ability to metabolize macromolecules into assimilable nutrients, to survive gastrointestinal-like conditions and exert probiotic effects. Based on current results, *B. cereus* F20, *B. benzoevorans* F21 and *B. tequilensis* F44 could serve as potential probiotic-starter cultures for high-quality fermented legume-based condiments production, whose consumption could allow their proliferation in the gastrointestinal tract where they can exert health benefits to the consumers.

## Conclusion

This study revealed that *Bacillus* spp. isolated from s*oumbala* display interesting probiotic and technological potential. These findings are supported by their ability to survive the conditions of gastrointestinal tract, antimicrobial activity and cell surface adhesion power that could favor their direct interaction. Based on these data, *B. cereus* F20, *B. benzoevorans* F21 and *B. tequilensis* F44 could serve as potential probiotic-starter cultures for the development and promotion of therapeutic and health-promoting fermented foods that may impact human health. Nevertheless, their effective use in humans may require further in vivo probiotic studies.

## Data Availability

All data generated and/or analyzed during the current study are included in this article. The accession numbers for the six Bacillus strains named B54 (MZ773905), F20 (MZ773907), F21 (MZ773908), F24 (MZ773909), F26 (MZ773911) and F44 (MZ773913) are available in NCBI.

## Abbreviations

BHI: Brain hearth infusion
ACS: Hydrocarbon solvents
AFF: Alkaline fermented foods
GRAS: Generally recognized as safe
PGA: Poly-γ-glutamic acid
FDA: Food and drug administration
GIT: Gastrointestinal tract

## Footnotes

1 https://www.ezbiocloud.net/
